# When Otorrhea Hides Something Unexpected: A Challenging Case Report

**DOI:** 10.7759/cureus.102762

**Published:** 2026-02-01

**Authors:** Susana Pipa, Liliana Ribeiro, Diogo Portugal, Ana Raquel S Afonso, Bebiana Conde

**Affiliations:** 1 Pulmonology Department, Unidade Local de Saúde de Trás-os-Montes e Alto Douro, Vila Real, PRT; 2 Otolaryngology Department, Unidade Local de Saúde de Trás-os-Montes e Alto Douro, Vila Real, PRT

**Keywords:** case reports, extrapulmonary tuberculosis, mastoiditis, mycobacterium tuberculosis infection, suppurative otitis media, tuberculosis

## Abstract

Tuberculosis remains a major global health concern, predominantly in low and middle-income countries. Tuberculous otitis media (TOM) is a rare form of extrapulmonary tuberculosis, representing a small fraction of tuberculosis and chronic suppurative otitis media cases. The nonspecific and indolent presentation of TOM, besides its rarity, often results in delayed diagnosis, mainly in regions with a low tuberculosis prevalence, with increased risk of irreversible complications.

We report a case of TOM in a 36-year-old Portuguese woman with a four-month history of right ear pain, otorrhea, obstruction, and progressive hearing loss. Despite multiple evaluations and empirical antibiotic treatments, symptoms worsened. Otoscopic assessment at our centre revealed chronic necrotizing otitis media with tympanic membrane perforation. Computed tomography demonstrated extensive right-sided mastoid and middle ear opacification without bone erosion. Audiometry confirmed moderate conductive hearing loss, and the patient underwent mastoidectomy and ossiculoplasty. Histopathology showed necrotizing inflammation, but microbiological studies were not performed initially.

Two weeks postoperatively, the patient developed respiratory and constitutional symptoms. Chest imaging revealed a cavitary lesion in the right lower lobe. Although initial direct and PCR sputum studies were negative, bronchoscopy confirmed *Mycobacterium tuberculosis *complex (MTC) through acid-fast bacilli staining and DNA testing. Retrospective PCR analysis of the ear surgical specimen also identified MTC, establishing the diagnosis of TOM with bone involvement. First-line antituberculosis therapy was initiated with rifampicin, isoniazid, pyrazinamide, and ethambutol. The patient completed 12 months of treatment with progressive clinical improvement, full restoration of hearing, and no residual otologic or systemic sequelae.

Due to its rarity and similarity to other chronic otologic infections, TOM is often misdiagnosed, leading to delays in correct diagnosis. Definitive diagnosis generally depends on histopathology and mycobacterial culture of mastoidectomy specimens. This case underscores the importance of maintaining a high index of suspicion for TOM in patients with chronic or atypical otitis media unresponsive to conventional therapy, especially in the presence of granulomatous or necrotizing features or relevant epidemiological exposures. Early recognition and appropriate management are essential to prevent irreversible complications and optimize patient outcomes.

## Introduction

Tuberculosis (TB) remains a major global health concern, responsible for about 1.3 million deaths annually, predominantly in low and middle-income countries [1,2]. Extrapulmonary TB accounts for 15-20% of TB cases [3]. Tuberculous otitis media (TOM) is an extremely rare extrapulmonary TB form, representing only 0.1% of TB cases and 0.04-0.9% of chronic suppurative otitis media [3], although recent studies suggest an incidence of up to 3.6% [4]. It usually follows an indolent course, classically presenting with painless otorrhea and multiple tympanic membrane perforations, though facial nerve palsy, progressive conductive hearing loss and granulation tissue in the middle ear and mastoid are also common features [3,5]. However, chronic otorrhea and tympanic membrane perforation are nonspecific findings shared with chronic suppurative otitis media of more common etiologies, which makes TOM particularly challenging to diagnose [4].

In a retrospective study comparing high-resolution temporal bone CT findings in tuberculous versus non-tuberculous otomastoiditis, no pathognomonic imaging features of TOM were identified. However, the authors concluded that certain radiologic findings may be suggestive of the diagnosis and could aid in early recognition [6]. These include soft-tissue density occupying the entire middle ear cavity, preservation of mastoid air cells without sclerotic changes, mucosal thickening of the bony external auditory canal, and extension of soft tissue into the external auditory canal, without erosion of the scutum.

Histopathological examination remains a reliable diagnostic method, together with microbiological analysis, including mycobacterial culture and molecular techniques, and early detection and appropriate management are determinant for improving outcomes and avoiding disease progression and sequelae [3].

Although some TOM case reports have recently been published, those originate mainly from countries with a high prevalence of TB [5,7]. Recent systematic reviews and European case series confirm the scarcity of TOM reports in Europe, with only isolated cases and small series published over the past 25 years [8,9]. The indolent course of TOM and its rarity in these populations often result in delayed diagnosis and increased risk of irreversible complications, such as permanent hearing loss or facial nerve paralysis [8].

This delayed recognition of TOM in low-prevalence European contexts (mean 13.6 months) makes it important to remind clinicians of this entity [8]. By exposing this clinical case, we intend to emphasize the importance of a detailed epidemiologic anamnesis and the need to consider TB, particularly extrapulmonary TB, in the event of a prolonged, unresponsive infection and granulomatous or necrotizing changes, which may require a high index of suspicion.

This case was previously presented as a poster at the XXIX Northern Congress of Pulmonology and the XXXV Galaico-Duriense Meeting, which occurred between the 24th and 26th of March, 2022.

## Case presentation

A 36-year-old Portuguese woman, with a history of allergic rhinitis and sporadic tobacco and inhaled drug use, presented with a four-month history of right ear pain, obstruction, otorrhea, and progressive hearing loss. She had worked in several American and Asian countries in the preceding five years and reported contact with a coworker diagnosed with TB in Thailand, three years earlier, but had never been screened.

**Figure 1:**
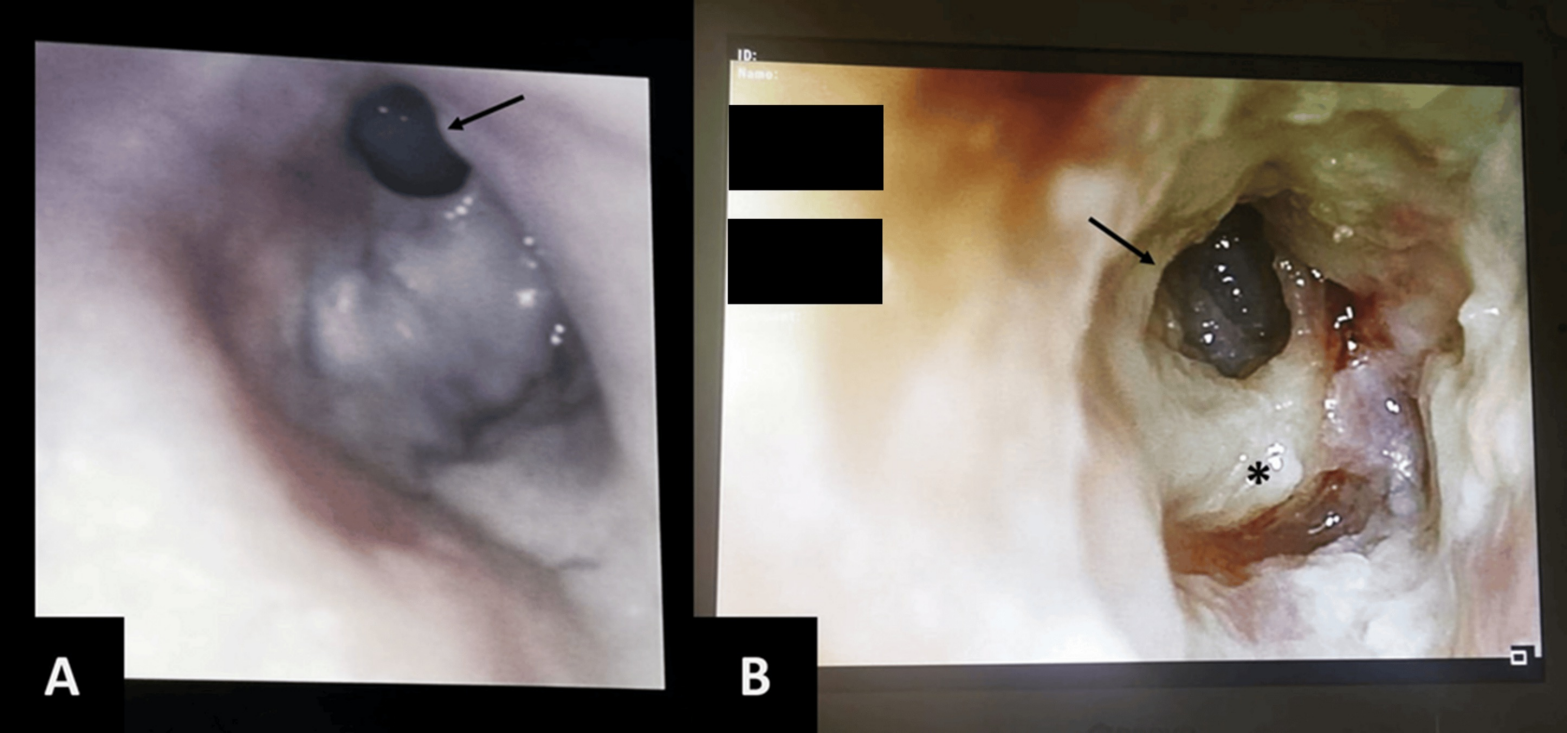
Otoscopy of the right ear. A. The image shows anterosuperior tympanic perforation (arrow) reported in April; B. The image shows evident progression, with enlargement of anterosuperior tympanic perforation (arrow) and necrotic remains of the tympanic membrane (*) reported in July.

Despite evaluation by several otorhinolaryngologists and multiple antibiotic courses, she showed no symptomatic improvement and progressive worsening of the ear alterations over several months (Figures 1A, 1B).

She was then observed by an otorhinolaryngologist at our centre, who identified chronic necrotising right otitis media, with tympanic perforation in both the anterosuperior and posterior quadrants, as well as necrotic remnants of the tympanic membrane.

**Figure 2:**
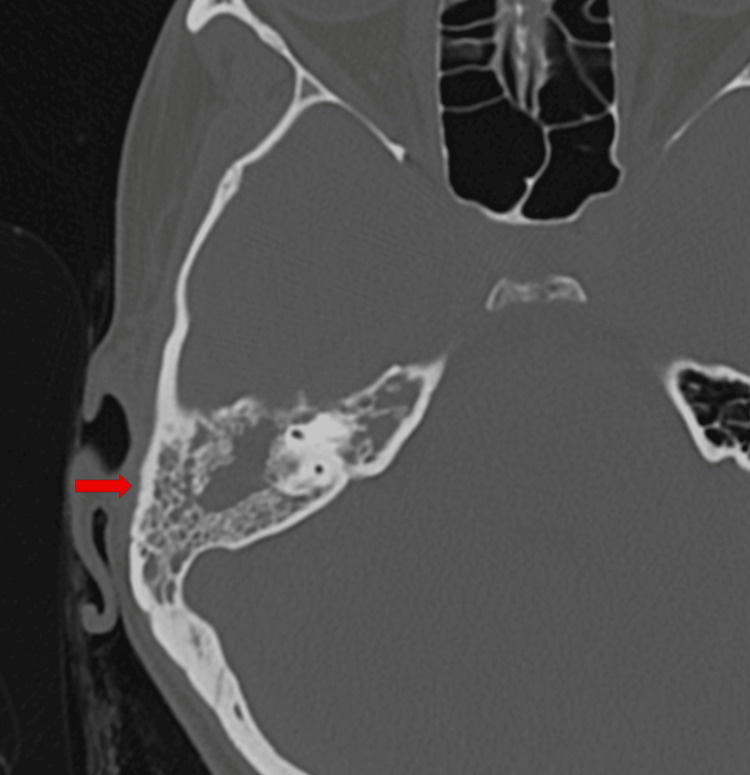
Ear CT scan evidencing complete opacification of the right mastoid cells (arrow). CT: computed tomography.

Ear CT scan showed complete opacification of the mastoid cells, with partial extension to the mastoid antrum and epitympanum, bilateral pneumatization and opacification of the right petrous apex, partial opacification of the mesotympanum and hypotympanum and thickening and slight retraction of the tympanic membrane, without bone erosion of the ossicular chain, mastoid cortex, or mastoid trabeculae (Figure 2). These imaging findings partially overlap with previously reported CT features suggestive of TOM [6], particularly the extensive soft-tissue involvement of the middle ear with preservation of the ossicular chain and mastoid bone, without evidence of erosion or sclerosis.

An audiogram evidenced moderate conductive hearing loss in the right ear (pure-tone average of 48 dB HL), and she was promptly submitted to right mastoidectomy and ossiculoplasty.

**Figure 3:**
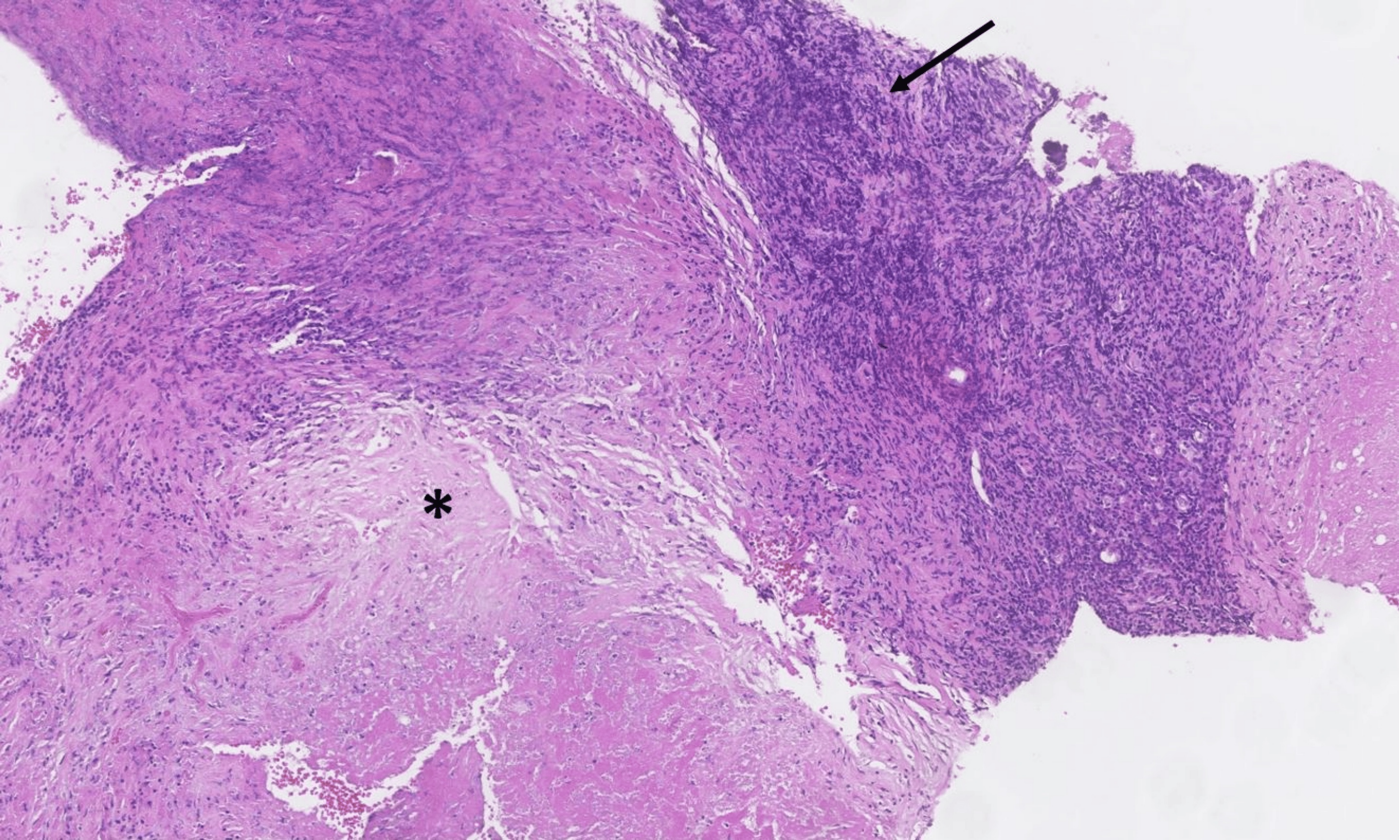
Histopathological findings from the fragments of tympanic cavity and mastoid (H&E, x10). The image demonstrates flaps of granulation tissue (arrow) and necrotic tissue (*), compatible with the origin of a necrotizing inflammatory lesion.

The histopathological examination of the fragments of the tympanic cavity and mastoid showed flaps of granulation and necrotic tissue, some related to small bone lamellae, compatible with a necrotizing inflammatory lesion (Figure 3).

**Figure 4:**
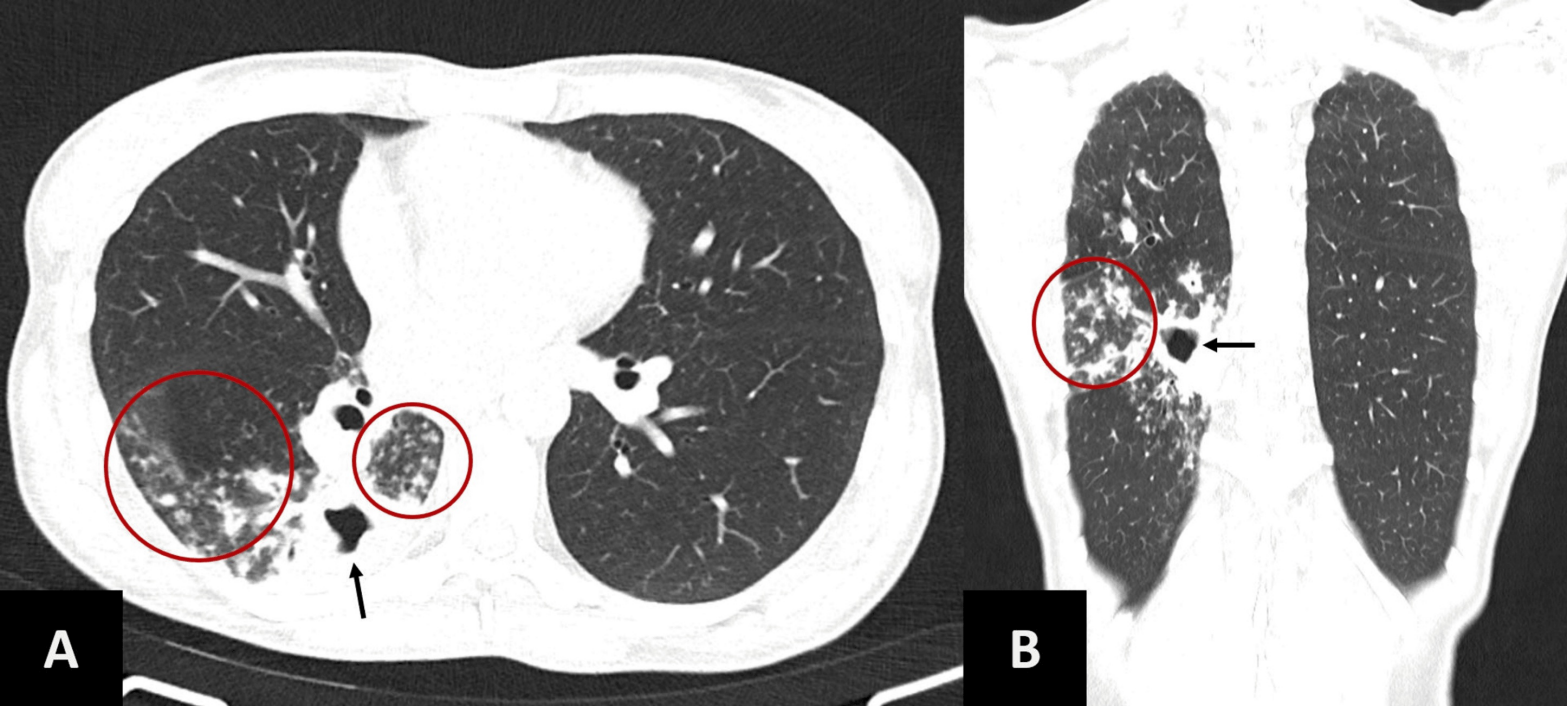
Chest CT scan A) Axial view and B) Coronal view. Scans evidencing a cavitated lesion in the upper segment of the right lower lobe (arrows) and ground glass infiltrates around the lesion (circles) CT - Computed tomography

Two weeks postoperatively, the patient developed a persistent cough, constitutional symptoms with involuntary three-kilogram weight loss and thoracalgia, which prompted clinical evaluation at an emergency department from another hospital. Chest X-ray showed a right hilar opacity, but initial sputum smears and DNA testing were negative for *Mycobacterium tuberculosis* complex (MTC). She was empirically medicated with amoxicillin/clavulanate and azithromycin for community-acquired pneumonia, with partial improvement at first, but after recurrence of symptoms, a chest CT scan was performed and revealed a cavitated lesion in the upper portion of the right lower lobe (Figures 4A, 4B), raising suspicion for tuberculosis.

**Figure 5:**
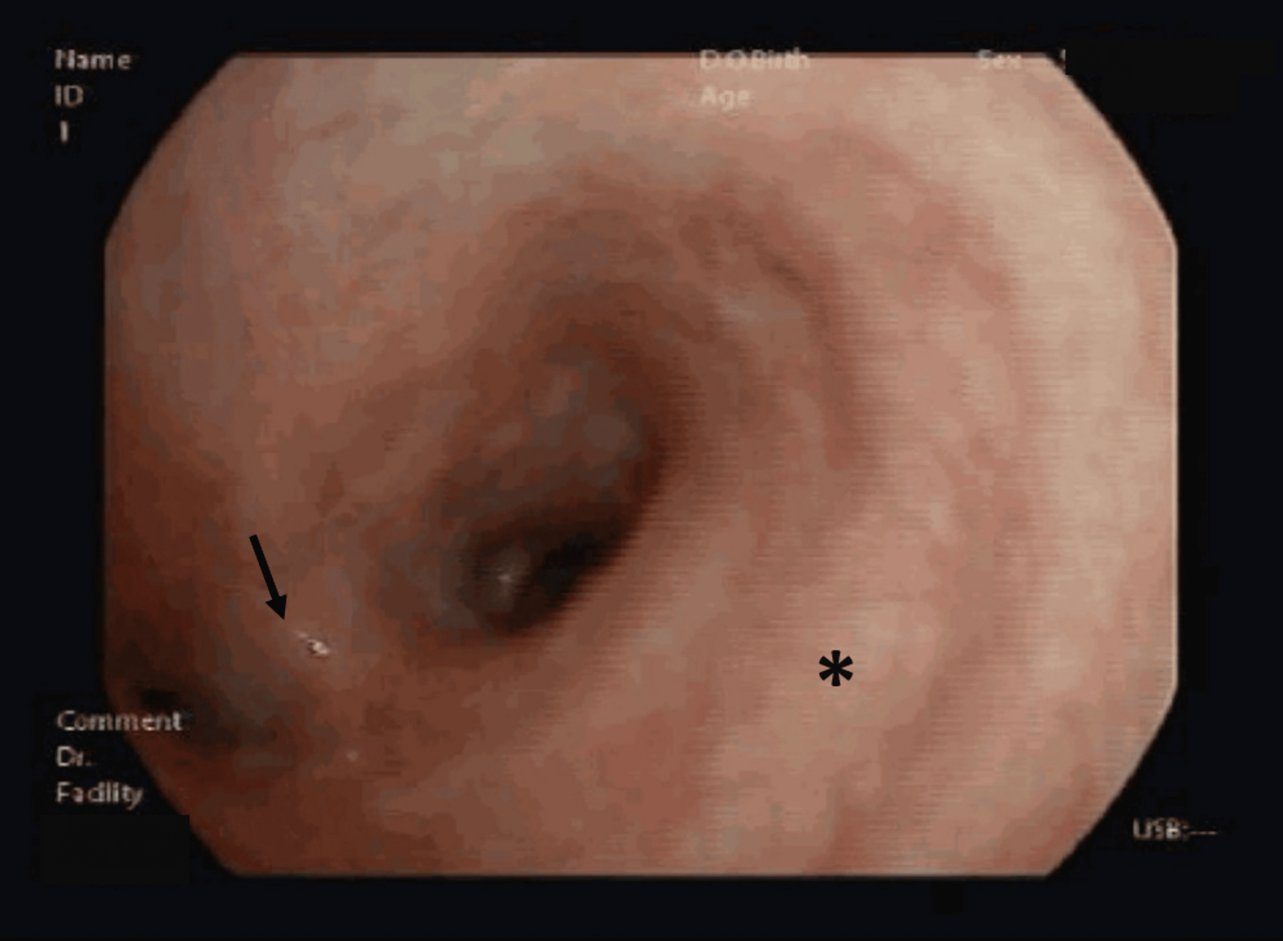
Bronchoscopic view of the right bronchial tree. The image demonstrates global mucosal oedema, with slight enlargement of the division spurs. The right main bronchus (*) and the right upper lobe spur (arrow) are identified.

The patient was forwarded to the Pulmonology emergency department. At admission, she was apyretic, without respiratory insufficiency and presenting crackles in the middle third of the right hemithorax at pulmonary auscultation. The sputum culture from the previous evaluation was still being processed at another centre and was later found to be contaminated. Bronchoscopy demonstrated the right bronchial tree globally oedematous, with slight enlargement of the division spurs (Figure 5); aspirate tested positive for acid-fast bacilli and MTC DNA, without rifampicin or isoniazid resistance. HIV testing was negative. First-line quadruple therapy was initiated with rifampicin, isoniazid, ethambutol and pyrazinamide.

Retrospective testing of the surgical specimen confirmed the presence of MTC DNA, establishing TOM with bone involvement (there was no initial mycobacteriological study). Ear evolution was favourable, with neo tympanum viability after six months and significant hearing restoration, with audiometry within the normal range bilaterally (pure-tone average of 19 dB HL on the right ear and 9 DB HL on the left). The patient completed 12 months of therapy with progressive clinical recovery.

In summary, we have a patient with a non-responsive chronic otitis media, with expanding tympanic perforation and necrotizing features. The surgical specimen evidenced granulation and necrotic tissues; however, microbiological analysis was not performed at first. She later developed a cough and systemic constitutional symptoms, which led to both the acknowledgement of previous tuberculosis contact and the diagnosis of cavitated pulmonary tuberculosis. The presence of MTC DNA in the surgical specimen was confirmed only retrospectively, at which point the diagnosis of TOM was established (Table 1).

**Table 1:** Timeline table detailing findings and procedures. CT: computed tomography; dB HL: decibels hearing level; ED: emergency department; MTC: *Mycobacterium tuberculosis *complex; H: Isoniazid, R: Rifampicin; Z: Pyrazinamide; E: Ethambutol.

Year/Month	Clinical Events	
2018	Risk contact with a case of tuberculosis in Thailand; no screening.	
2021		
March	Initial symptoms: right ear pain and obstruction with hearing loss	Multiple antibiotic courses
April	Otoscopy: anterosuperior tympanic perforation	
Early July	Otoscopy: evident progression with enlargement of anterosuperior tympanic perforation and necrotic remains of the tympanic membrane	
Late July	Otoscopy: multiple tympanic perforations and necrotic remains of the tympanic membrane. Ear CT scan: complete opacification of the mastoid cells, thickening and slight retraction of the tympanic membrane. Audiogram: moderate conductive hearing loss (pure-tone average of 48 dB HL)	
Early August	Surgery: mastoidectomy and ossiculoplasty. Histopathologic examination: inflammatory necrotic lesion (granulation and necrotic tissue). No mycobacteriological study of the surgical specimen was performed	
Mid August	Irritative cough, weight loss, astheny + chest pain prompted ED evaluation. Chest X-ray showed right hilar opacity. Sputum smear and DNA testing were negative for MTC. Contaminated culture. Empirical amoxicillin/clavulanate and azithromycin	
September	Re-aggravation of symptoms prompted a chest CT scan: cavitated lesion in the right lower lobe. Forwarded to pulmonology ED. Acknowledgement of previous risk contact with tuberculosis. Bronchoscopy performed, and bronchial aspirate tested positive for acid-fast bacilli and MTC DNA; no resistance to R or H. Initiated HRZE. Retrospective testing of the surgical specimen: presence of MTC DNA	
2022, March	Otoscopy: neo tympanum viability. Audiometry of the right ear within the normal range (pure-tone average of 19 dB HL)	

## Discussion

TOM is an uncommon extrapulmonary TB manifestation, particularly in high-income countries. A recent European review confirms that TOM remains a diagnostic challenge due to non-specific symptoms and frequent misdiagnosis as chronic suppurative otitis media. The most common presenting features in European cases are chronic otorrhea (100%), hearing loss (86%), eardrum perforation (44%), ear pain (30%), and facial paralysis (28%). The majority of cases were unilateral (76.7%), although 10 patients (23.3%) presented bilateral disease [8].

Diagnosis is often difficult, especially when otologic symptoms precede pulmonary ones. From the 43 cases identified in this European review, only seven patients were confirmed to have TB disease in other locations [8]. Therefore, direct implantation through the external auditory canal and tympanic membrane should be considered a possible route of spread of TB, and it is crucial to consider TOM even in the absence of other signs of TB. Diagnostic confirmation is most often achieved by biopsy obtained during mastoidectomy (79%), as microbiological cultures and PCR of ear exudate have limited sensitivity and are usually not requested due to low initial suspicion in most cases [8,9].

However, in cases of progressive, unresponsive chronic suppurative otitis media of unclear etiology requiring surgical intervention, microbiological studies, including mycobacterial cultures and MTC DNA, should be performed, as they may allow for earlier diagnosis of TOM. In our case, this approach could have enabled diagnosis approximately one and a half months earlier and initiation of treatment at an earlier stage of the disease. Even in the absence of initial mycobacteriological testing, the presence of necrotizing or granulomatous changes on histopathological examination should prompt consideration of TOM.

In Aguilera-Franco’s review, hearing loss was the most frequent sequela after infection resolution (n=28; 65.12%), while permanent facial paralysis occurred in three patients (6.98%), which, despite the lower incidence, is not negligible due to its severity. Importantly, the risk of sequelae was closely related to diagnostic delay, which ranged from one to 72 months (mean 13.6 months) [8].

In our case, the time from initial symptoms to diagnosis was six months, well below the reported mean [8]. However, this interval might have been shorter with earlier microbiological investigation, or longer in the absence of respiratory symptoms that ultimately raised suspicion of tuberculosis, which illustrates how strongly the diagnostic timeline depends on clinical awareness. Fortunately, our patient recovered without sequelae.

## Conclusions

This case highlights the need for heightened clinical suspicion for TOM in patients with chronic or atypical otitis media unresponsive to conventional therapy, particularly if necrotizing or granulomatous changes are present. Early clinical suspicion, a thorough epidemiological history, and integration of clinical, laboratory, and radiological findings, together with prompt microbiological and histopathological evaluation, are critical for timely diagnosis and optimal management. It holds particular clinical value for two reasons. First, it illustrates disease progression through otoscopic images, which may facilitate recognition in clinical practice. Secondly, it demonstrates the potential utility of surgical specimen re-evaluation in cases with suspicious histopathological features, especially in the absence of an initial microbiological study.
